# Subcutaneous transplantation of human embryonic stem cells-derived pituitary organoids

**DOI:** 10.3389/fendo.2023.1130465

**Published:** 2023-03-02

**Authors:** Hiroo Sasaki, Hidetaka Suga, Kazuhito Takeuchi, Yuichi Nagata, Hideyuki Harada, Tatsuma Kondo, Eiji Ito, Sachi Maeda, Mayu Sakakibara, Mika Soen, Tsutomu Miwata, Tomoyoshi Asano, Hajime Ozaki, Shiori Taga, Atsushi Kuwahara, Tokushige Nakano, Hiroshi Arima, Ryuta Saito

**Affiliations:** ^1^ Department of Neurosurgery, Graduate School of Medicine, Nagoya University, Nagoya, Japan; ^2^ Department of Endocrinology and Diabetes, Graduate School of Medicine, Nagoya University, Nagoya, Japan; ^3^ Regenerative & Cellular Medicine Kobe Center, Sumitomo Pharma Co., Ltd., Kobe, Japan; ^4^ Environmental Health Science Laboratory, Sumitomo Chemical Co., Ltd., Osaka, Japan

**Keywords:** ACTH, organoid, pituitary, regenerative medicine, subcutaneous transplantation

## Abstract

**Introduction:**

The pituitary gland, regulating various hormones, is central in the endocrine system. As spontaneous recovery from hypopituitarism is rare, and exogenous-hormone substitution is clumsy, pituitary replacement *via* regenerative medicine, using pluripotent stem cells, is desirable. We have developed a differentiation method that in mice yields pituitary organoids (POs) derived from human embryonic stem cells (hESC). Efficacy of these POs, transplanted subcutaneously into hypopituitary mice, in reversing hypopituitarism was studied.

**Methods:**

hESC-derived POs were transplanted into inguinal subcutaneous white adipose tissue (ISWAT) and beneath dorsal skin, a relatively avascular region (AR), of hypophysectomized severe combined immunodeficient (SCID) mice. Pituitary function was evaluated thereafter for ¾ 6mo, assaying basal plasma ACTH and ACTH response to corticotropin-releasing hormone (CRH) stimulation. Histopathologic examination of organoids 150d after transplantation assessed engraftment. Some mice received an inhibitor of vascular endothelial growth factor (VEGF) to permit assessment of how angiogenesis contributed to subcutaneous engraftment.

**Results:**

During follow-up, both basal and CRH-stimulated plasma ACTH levels were significantly higher in the ISWAT group (*p* < 0.001 – 0.05 and 0.001 – 0.005, respectively) than in a sham-operated group. ACTH secretion also was higher in the ISWAT group than in the AR group. Histopathologic study found ACTH-producing human pituitary-cell clusters in both groups of allografts, which had acquired a microvasculature. POs qPCR showed expression of angiogenetic factors. Plasma ACTH levels decreased with VEGF-inhibitor administration.

**Conclusions:**

Subcutaneous transplantation of hESC-derived POs into hypopituitary SCID mice efficaciously renders recipients ACTH-sufficient.

## Introduction

The pituitary gland, an important endocrine center, regulates homeostasis *via* various hormones. The anterior pituitary lobe secretes adrenocorticotropic hormone (ACTH), growth hormone, thyroid-stimulating hormone, luteinizing hormone, follicle-stimulating hormone, and prolactin. The posterior pituitary lobe secretes oxytocin and vasopressin. These hormones support a wide variety of physiological functions. Deficiency of pituitary hormones thus can cause severe systemic disease, variably manifest (1). For example, ACTH deficiency can cause adrenocortical insufficiency, resulting in impaired consciousness, electrolyte imbalance, hypotension, and compromised immunity, which can at worst be fatal (2). The most common cause of hypopituitarism is pituitary adenoma (3, 4). Other non-pituitary tumors, such as craniopharyngioma or meningioma, also can cause hypopituitarism. Non-neoplastic causes include Sheehan syndrome, elevated intracranial pressure with empty sella syndrome, traumatic brain injury, aneurysmal subarachnoid hemorrhage, and hypophysitis; some instances are idiopathic (3, 4). Present treatment for hypopituitarism is, with rare exceptions (5), limited to administration of hormones identified as deficient. Dosage adjustment is difficult. Irregular administration – patients forget! – also poses problems, lessening utility (2). If pituitary-gland tissue derived from pluripotent stem cells (PSCs) could be deployed clinically, however, these complications of hormone replacement therapy might be eliminated.

We have successfully established efficient differentiation of human embryonic stem cells (hESC) and human induced pluripotent stem cells (hiPSC) into pituitary organoids (POs) *in vitro* (6, 7), working from a three-dimensional differentiation method using mouse ESC (7). Transplanting hESC-derived POs under the renal capsule of hypopituitary mice improves activity levels and mortality (6, 8). Differentiation of hESC-derived POs under feeder-free conditions is under way (9); clinical application is in the offing. However, several issues remain. Among them is determination of the site and method of transplantation. We hitherto have transplanted PSCs-derived POs into mice using a renal subcapsular site, but the trans-retroperitoneal approach required is substantially invasive. Graft removal from this site, should tumor improbably develop (10, 11), and renal injury with insufficiency also are concerns. Site and method of transplantation thus require refinement. This study accordingly sought to identify an easy, relatively non-invasive, and – in case of tumorigenesis – extirpation-accessible approach for PO graft placement.

## Materials and methods

### Maintenance and differentiation culture of hESCs

hESCs (KhES-1) were provided by RIKEN BioResource Center and were used in accordance with the hESC research guidelines of the Japanese government. All experimental protocols and procedures were approved by the Ethics Committee of Nagoya University Graduate School of Medicine (approval ES-001). Maintenance and differentiation culture of hESCs was performed as described (6–8, 12) with modifications (9) (**Supplementary Figure 1**). POs harvested 100-200d after differentiation were used.

### Mice and hypophysectomy

All animal experiments were approved by the Animal Experimentation Committee of the Nagoya University Graduate School of Medicine and were performed in accordance with institutional guidelines for animal care and use. Severe combined immunodeficient (SCID) male mice aged 8-9wk (C.B-17/Icr-Hsd-Prkdc^scid^, Japan SLC, Shizuoka, Japan) underwent transaural hypophyseal ablation (13). Mice were anaesthetized with intraperitoneal (i.p.) injection of a mixture of 3 agents (medetomidine 0.75mg/kg, midazolam 4mg/kg, butorphanol 5mg/kg) (14) and pituitary tissue was aspirated from the sella turcica *via* the auditory meatus using a needle (KN-390, Natsume Seisakusyo, Tokyo, Japan) fitted to a 1ml syringe containing 0.2 ml saline. After the procedure, mice were injected with the medetomidine antagonist atipamezole (0.75mg/kg i.p.). Since hypopituitary SCID mice are sickly, they were bred in a clean environment in the P2A laboratory, a newly constructed animal facility at our university. Their cages were changed twice a week. Complete hypophysectomy was confirmed post-mortem in all mice used (**Supplementary Figure 2**).

Blood collection and ACTH determination

**Figure 1:**
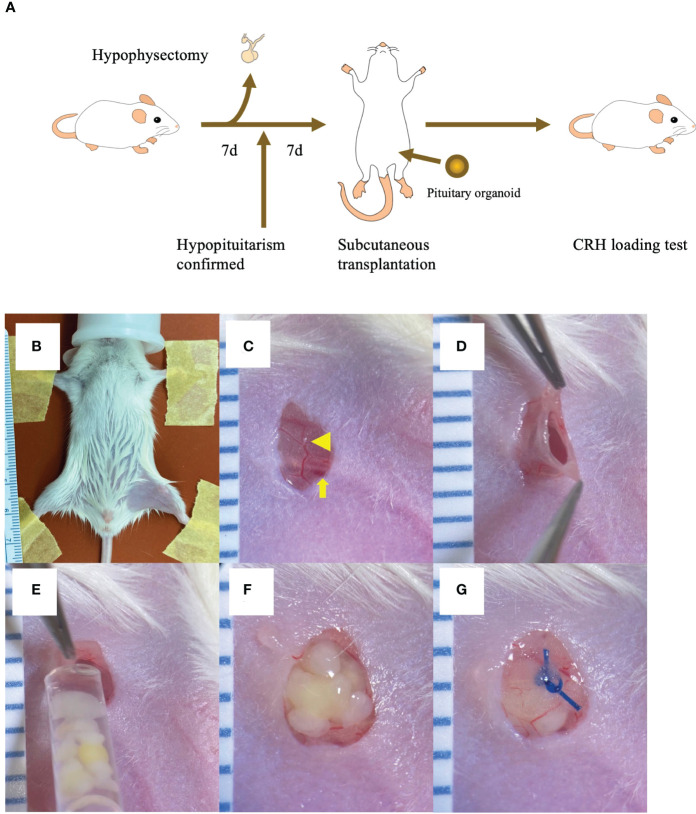
Inguinal subcutaneous white adipose tissue (ISWAT) transplantation of hESC-derived pituitary organoids (POs) **(A)** Schema, mouse handling protocol (hypophyseal ablation, confirmation of hypopituitarism, POs transplantation, and allograft-function testing). **(B)** Shaved left inguinal area, restrained supine mouse under inhalation anesthesia. **(C)** Vessel in filmy adipose tissue (arrowhead) overlying femoral vein and artery (arrowhead) viewed through 4mm vertical skin incision. **(D)** Pocket created in ISWAT. **(E)** POs emplacement into pocket *via* syringe fitted with wide-bore tip. **(F)** Emplaced POs. **(G)** Nylon suture closure of adipose tissue over POs. Scale intervals, 1mm.

ACTH levels were assessed as a biomarker of pituitary function. Blood samples were collected by tail transection between 1300 and 1700, with sampling before and 1h after administration of human CRH (2μg/kg, i.p.; Tanabe, Osaka, Japan). Plasma was separated from blood samples by centrifugation at 1,000 x g for 15min, 4°C. Plasma ACTH assay used an ACTH ELISA kit (MD Bioproducts, Oakdale, MN) reactive against human and mouse ACTH, with solution absorbance (ACTH concentration) read using Cytation 5 (Biotek, Winooski, VT). CRH loading tests were conducted 1wk after hypophysectomy and 1/~4wk after PO transplantation, including in sham-operated mice, until 6mo later (**Figure 1A**). Blood samples were collected repeatedly from the same mice. To prevent adrenal crisis, all mice received intramuscular dexamethasone, 0.2mg/0.61ml, after each blood collection. Mice with plasma ACTH levels < 10pg/ml after CRH stimulation were classed as hypopituitary and used as subjects.

### Determination of PO spontaneous ACTH secretion *in vitro*

Five POs were incubated at 37°C for 72h in 2.5ml cell culture medium (Iscove’s modified Dulbecco’s medium, Sigma-Aldrich, St. Louis, MO; Ham’s F12, Thermo Fisher Scientific, Waltham, MA (1:1); 1% GlutaMAX, Thermo Fisher Scientific; 1% Chemically Defined Lipid Concentrate, Thermo Fisher Scientific; 450 µM 1-thioglycerol, Sigma-Aldrich; and 20% KnockOut Serum Replacement, Thermo Fisher Scientific). Culture supernatants were collected. ACTH concentrations in supernatants were determined using an electrochemiluminescence immunoassay (ECLIA) kit (SRL, Tokyo, Japan) employed clinically in Japan.

### Transplantation methods

Mice were anesthetized with isoflurane and placed supine. In inguinal subcutaneous white adipose tissue (ISWAT) transplantation, the left inguinal area was shaved. A 4mm vertical skin incision was made and a pocket in subcutaneous adipose tissue was created, with placement of 5 POs harvested from cell culture medium into the pocket using a wide-bore tip under microscopy. Nylon-suture closure over the POs was followed by skin closure (**Figures 1B–G**). In the sham-operated group, a vertical skin incision was made in the left inguinal region, a small pocket was created in the subcutaneous fat, and surgical wound closure was performed without PO transplantation. In the avascular region (AR) transplantation group, a vertical skin incision was made in the left dorsal skin, with placement of 5 POs. After these manipulations, mice received intramuscular dexamethasone (0.2mg/0.61ml).

### Evaluation of mice transplanted with POs

Weight was followed and rate of weight loss was evaluated. Activity testing used a running wheel device (ENV-044; Med Associates, Georgia, VT).

### Histological assessment

Transplanted cell aggregates, skin, and fat, from SCID mice were fixed in 10% formalin, dehydrated for paraffin infiltration, and sectioned by sliding microtome. Sections at 5µm were stained with hematoxylin and eosin (H&E) or subjected to immunofluorescence microscopy for various antigens with nuclear 4′,6-diamidino-2-phenylindole counterstaining. Antigen targets included ACTH (mouse, 1:200, 10C-CR1096M1; Fitzgerald), LHX3 (LIM homeobox protein 3, rabbit, 1:3000, AS4002S; RIKEN custom), human nuclei (mouse, 1:1000, MAB4383; Millipore), E-cadherin (rat, 1:50, M108; Takara) and SMA (smooth-muscle actin, mouse, 1:200, M0851; DAKO).

RNA extraction and cDNA synthesis from POs and undifferentiated hESCs

RNA was extracted from POs and undifferentiated hESCs using the RNeasy Mini Kit (Qiagen, Hilden, Germany) following manufacturer’s instructions. RNA quality was evaluated using TapeStation 4150 (Agilent Technologies, Santa Clara, CA). cDNA was synthesized using ReverTra Ace qPCR RT Master Mix with gDNA Remover (Toyobo, Osaka, Japan).

### Quantitative PCR

Quantitative PCR (qPCR) of 5 POs and 5 transplanted POs (differentiated from 10,000 ESCs/sample) used a LightCycler 480 system (Roche Diagnostics, Rotkreuz, Switzerland). Data were normalized to those for *GAPDH* as an endogenous control and determined using standard curve-based relative quantitation. Primers used were: *VEGFA*, forward 5’-CTGTCAGGGCTGCTTCTTC-3’, reverse 5’-TTGCTGTGCTTTGGGGATTC -3’; *VEGFB*, forward 5’-TTGACTGTGGAGCTCATGGG-3’, reverse 5’-TGTGTTCTTCCAGGGACATCT-3’; *VEFGC*, forward 5’-TGTTTTCCTCGGATGCTGGA-3’,reverse 5’-ACATTGGCTGGGGAAGAGTT-3’; *FGF2*, forward 5’-AGGAGTGTGTGCTAACCGTT-3’, reverse 5’-CAGTTCGTTTCAGTGCCACA-3’; *ANGPT2*, forward 5’-TGACTGCCACGGTGAATAAT-3’, reverse 5’-CGTGTAGATGCCATTCGTGG-3’; *GAPDH*, forward 5’-CATCACTGCCACCCAGAAGACTG-3’, reverse 5’-ATGCCAGTGAGCTTCCCGTTCAG -3’. These primer sequences do not cross-react between human and mouse genes. qPCR was performed in POs and contralateral ISWAT. As thus assayed, expression of these genes was clearly lower in mouse ISWAT than in POs (**Supplementary Figure 3**).

### Statistical analysis

All data were analyzed using IBM SPSS statistics software (version 28.0.0.0, IBM, Armonk, NY). Data are expressed as means ± standard error. Comparisons between groups were performed using Student’s *t*-test. Comparisons among groups were performed by one-way ANOVA with *post hoc* Tukey’s test. *P* values of < 0.05 (*), < 0.01 (**), and < 0.001 (***) were considered significant.

## Results

### Assessment of subcutaneous transplantation methods

Four methods were examined: Pre-vascularization of subcutaneous tissue using temporary placement of either a gelatin hydrogel sustained-release device containing basic fibroblast growth factor (FGF) with heparin (GEL) (15) or of a medically approved vascular access catheter (deviceless, DL) (16); graft siting in ISWAT (17); and graft siting in a relatively AR beneath dorsal skin. We hypothesized that blood supply would determine graft fate. AR siting served as a control for the 3 other options. Adipose tissue is inherently vessel-rich, while the GEL and DL pre-vascularization methods increase blood supply to the graft site. However, those methods require invasive manipulation 2wk or 1mo, respectively, before transplantation, potentially stressing hypopituitary mice. Plasma ACTH levels 1mo after transplantation of hESC-derived POs were highest in ISWAT-cohort mice (ACTH levels in POs culture medium 30100 pg/ml; all transplanted POs from the same lot). ISWAT transplantation thus appeared best (**Figure 1**, **Supplementary Figure 4**).

### Comparisons among ISWAT, sham-operated, and AR groups

ISWAT group (n=6), sham-operated group (n=6), and AR group (n=5) mice, as stated above, served as controls for higher-vascularity ISWAT work, with follow-up of plasma ACTH levels for 6mo. Pre-transplant basal plasma ACTH levels and CRH-stimulated plasma ACTH levels did not significantly differ among the 3 groups (ISWAT *vs.* sham *vs.* AR, basal; 4.6 ± 1.8 pg/ml *vs. 2.3* ± 1.6 pg/ml *vs.* 2.5 ± 1.1pg/ml, *p* = 0.27 – 0.92, stimulated; 1.7 ± 0.8 pg/ml *vs.* 1.7 ± 2.3 pg/ml *vs.* 7.2 ± 2.1 pg/ml, *p* = 0.06 – 0.72).

**Figure 2:**
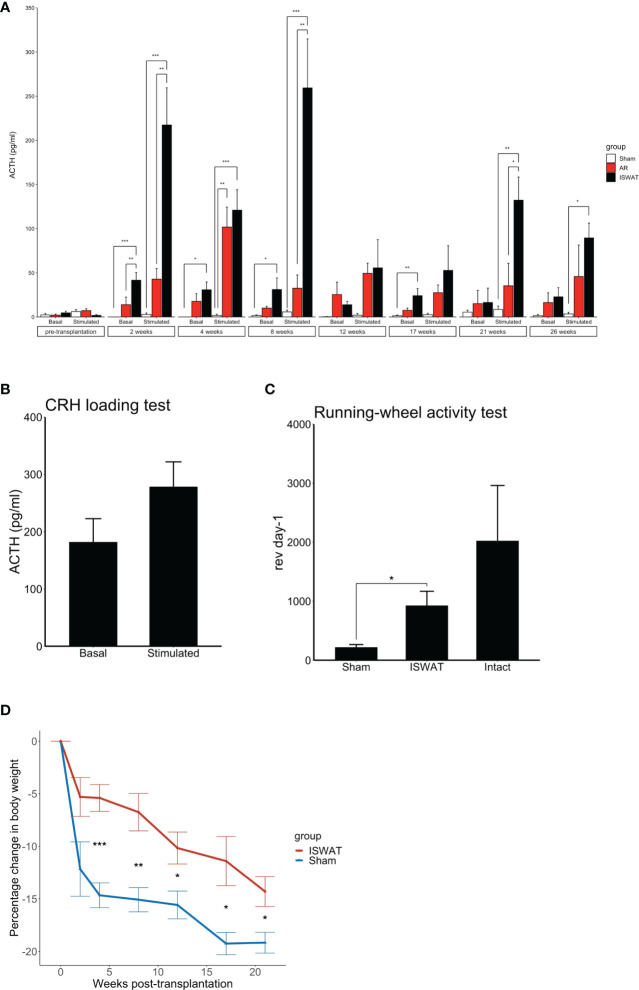
Evaluation of grafted mice All data presented as mean ± SEM. *p<0.05, **p<0.01, ***p<0.001. **(A)** Basal and CRH-stimulated serum ACTH levels in mice subjected to POs transplantation; sham-operated (Sham), avascular region (AR), and ISWAT cohorts. Sham, n=6. AR, n=5. ISWAT, n=6. Statistical assessment, one-way ANOVA with *post hoc* Tukey’s test. **(B)** CRH loading test in intact SCID mice. n=6. **(C)** Running-wheel activity test. Sham, n=3. ISWAT, n=3. Intact, n=3. Student’s *t*-test. **(D)** Percentage change in body weight, ISWAT and Sham mice. Percentage change in body weight, abscissa; time course, ordinate. Student’s *t*-test.

After transplantation, basal plasma ACTH levels (“basal”, **Figure 2A**) consistently were higher in the ISWAT group than in the sham-operated group. At 2, 4, 8, and 17wk after transplantation, ACTH values differed significantly between the groups (*p* < 0.001 – 0.05, **Figure 2A** *)*. CRH-stimulated plasma ACTH levels (“stimulated”, **Figure 2A**) also were higher in the ISWAT group than in the sham-operated group, with statistically significant differences between the groups at 2, 4, 8, 21, and 26wk (*p* < 0.001 – 0.005, **Figure 2A** *)*.

Pre-transplant ACTH secretion *in vitro*, as assessed by ACTH levels in POs culture medium, did not differ significantly between the ISWAT and AR groups (ISWAT *vs.* AR, 44416 ± 8435 pg/ml *vs.* 45800 ± 17291 pg/ml, p=0.876); *i.e.*, ACTH secretory capacity of transplanted POs was similar between groups. Basal plasma ACTH levels in intact mice were 182 ± 40.7 pg/ml and CRH-stimulated plasma ACTH levels were 278.6 ± 43.2 pg/ml (n=6, **Figure 2B**). After transplantation, plasma basal ACTH levels and CRH-stimulated ACTH levels were higher in the AR group than in the sham-operated group and lower than in the ISWAT group, with ISWAT plasma basal ACTH levels significantly higher than AR levels at 2wk (p = 0.009) and ISWAT plasma CRH-stimulated ACTH levels significantly higher than AR levels at 2, 8, and 21wk (p < 0.001 – 0.013). These results indicated that POs in subcutaneous tissues released ACTH more efficiently when implanted in well-vascularized sites such as adipose tissue than in non-vascularized sites.

Whilst running wheel testing found greater activity in the ISWAT group than in the sham-operated group, the ISWAT group was slightly less active than the intact group (**Figure 2C**). The rate of weight loss in the ISWAT group was modest by comparison with that in the sham-operated group, but still > 10% (**Figure 2D**).

### Macroscopic and histological findings after subcutaneous transplantation

**Figure 3:**
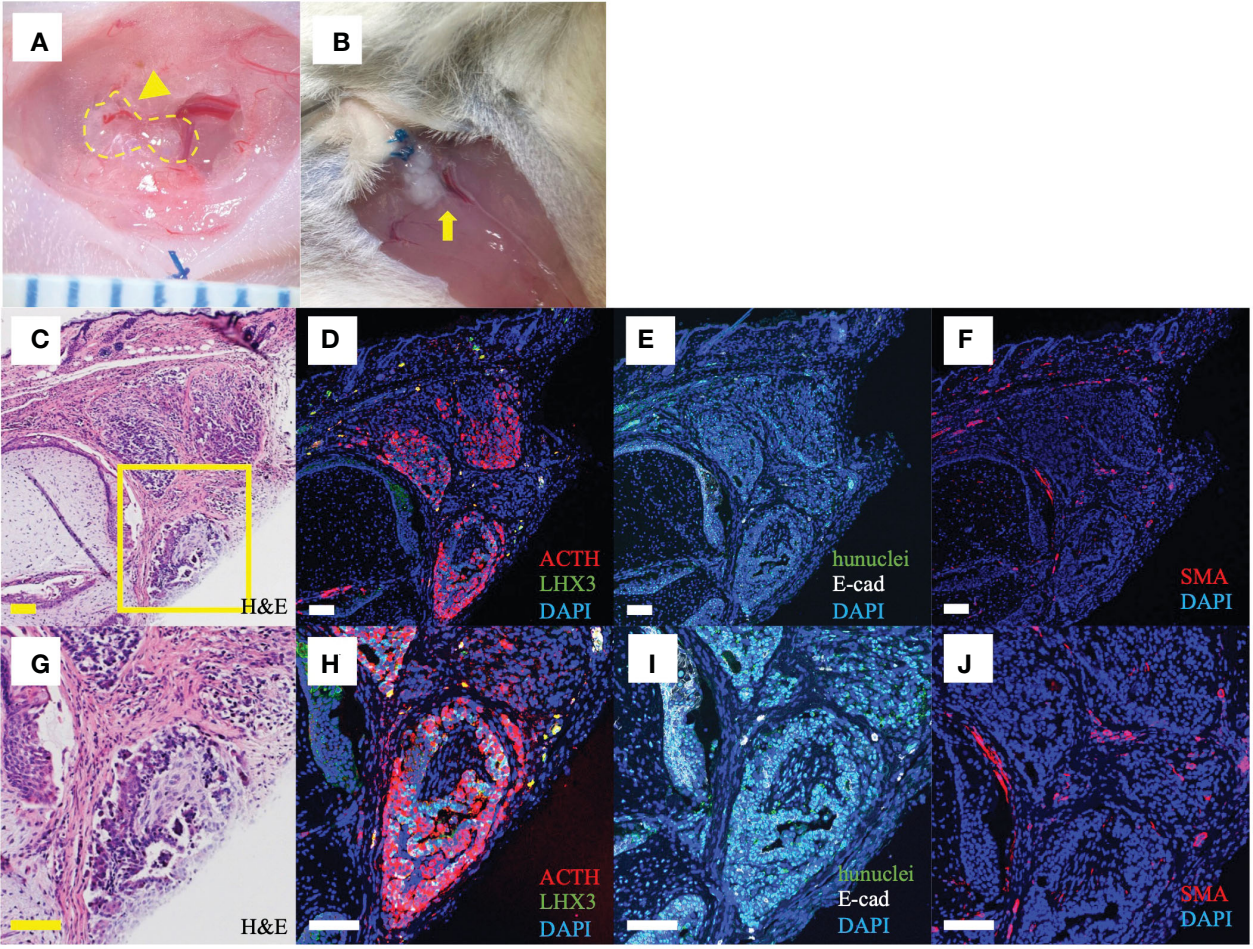
Macroscopic and histological findings after ISWAT transplantation **(A)** Macroscopic appearance, graft site, 4wk after transplantation. Dotted line, grafted POs. Arrowhead, vessel associated with graft. Scale intervals, 1mm. **(B)** Macroscopic appearance, 21wk after transplantation. Arrow, engrafted POs. **(C, G)** Skin and subjacent tissue including graft. Several POs are included in the section. Hematoxylin/eosin **(H, E)**. Yellow box, **(D–J)**, **(D–F, H–J)** Immunofluorescence photomicrographs, various antigens targeted. Counterstaining with 4,6-diamidine-2 -phenylindole dihydrochloride (DAPI). **(D, H)** Adrenocorticotropic hormone (ACTH, red) and LIM-homeobox protein (LHX3, green), a pituitary progenitor marker. **(E, I)** Human nuclei (hunuclei, green) and E-cadherin (E-cad, white), an oral ectoderm marker. **(F, J)** Smooth muscle actin (SMA, red), a vessel-wall marker. Scale bars uniformly 100 μm.

On macroscopy 4wk after ISWAT transplantation, graft neovascularization was apparent (**Figure 3A**). At 21wk, no adipose tissue was observed macroscopically, but the grafts appeared intact (**Figure 3B**). On microscopy of the skin and subjacent grafts harvested *en bloc* at 21wk, transplanted cell aggregates lay within subcutaneous tissue (**Figures 3C, G**). Fluorescence immunomicroscopy revealed that the grafts expressed ACTH; E-cadherin, a marker of oral ectoderm; and LHX3, a pituitary-progenitor marker (**Figures 3D, E, H, I**). Simultaneous expression of human nuclei indicated that the cells in question were transplanted hESC-derived pituitary cells (**Figure 3I**). Furthermore, SMA, a vessel-wall marker, was expressed as clusters around and within the grafts (**Figures 3F, J**), indicating neovascularization. These observations indicate that the hESC-derived POs engraft and function *in vivo*. To confirm by microscopy that POs were present was not possible in AR-group mice that secreted ACTH poorly, whereas in an AR-group mouse with relatively good ACTH secretion tissue with engrafted POs was found on H&E staining. Fluorescence immunomicroscopy also demonstrated reactivity, although fewer cells marked than in ISWAT material (**Supplementary Figure 5**).

### Promoting vascularization of hESC-derived POs

**Figure 4:**
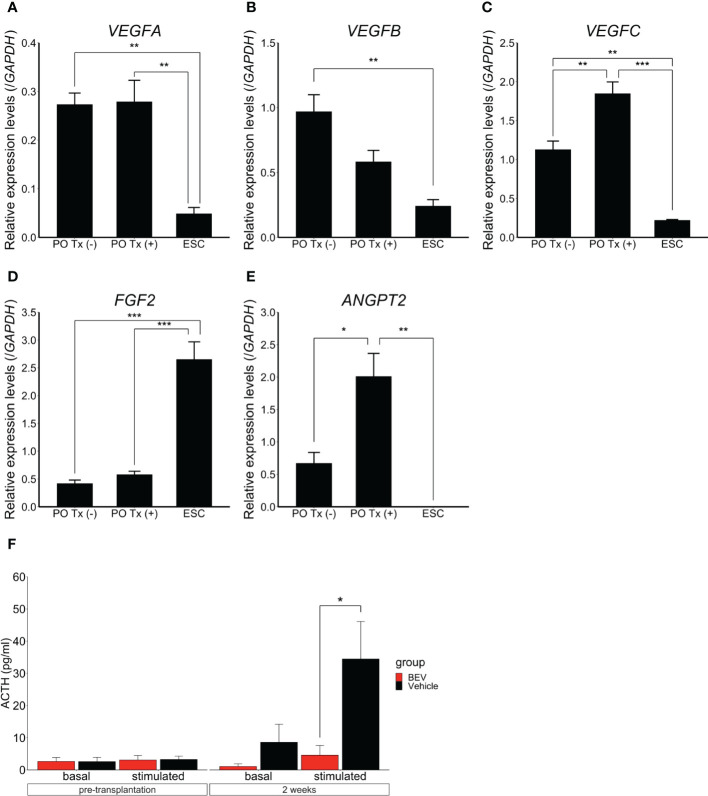
Expression and effects of angiogenic factors in hESC-derived POs All data presented as mean ± SEM. *p<0.05, **p<0.01, ***p<0.001. Quantitative PCR results, expression of *VEGFA* **(A)**, *VEGFB* **(B)**, *VEGFC* **(C)**, *FGF2* **(D)**, and *ANGPT2* **(E)** in POs without transplantation, transplanted POs (“with transplantation”), respectively PO Tx (-) and PO Tx (+), and undifferentiated ESC. Expression was normalized to that of *GAPDH*. PO Tx (-), n=3. PO Tx (+), n=3. ESC, n=3. Statistical assessment, one-way ANOVA with *post hoc* Tukey’s test. **(F)** Comparison of basal and CRH-stimulated serum ACTH levels with bevacizumab (BEV) or vehicle administration. BEV, n=9. Vehicle, n=9. Student’s *t*-test.

Acknowledging that hESC-derived POs function after subcutaneous transplantation, the question arose of how vascularization affects engraftment of POs transplanted subcutaneously. Vascular endothelial growth factor (VEGF), FGF2, and angiopoietin 2 (ANGPT2) are related to early-stage angiogenesis (18–20). We evaluated by qPCR whether the POs expressed these genes. Pre-transplant POs, POs harvested 12h after transplantation, and undifferentiated hESCs as controls were analyzed. Expression levels of *VEGFA*, *VEGFB*, *VEGFC*, and *ANGPT2* in the POs were significantly higher than those in undifferentiated hESCs. Expression levels of *VEGFC* and *ANGPT2* in particular rose after transplantation (**Figures 4A–E**). These results suggested that the POs themselves express angiogenic factors, which might promote engraftment into subcutaneous tissue.

Finally, to assess further the importance of angiogenesis in survival of subcutaneous POs grafts, we examined POs function *in vivo* by administering bevacizumab, a VEGF inhibitor (2mg/kg, i.p., 2x/wk for 2wk (21); Selleck, Houston, TX). Hypopituitary SCID mice were divided after ISWAT transplantation into a bevacizumab administration group (n=9) and a vehicle administration group (n=9). Whilst ACTH levels in the culture medium of POs used in grafting did not differ significantly between the groups (bevacizumab *vs.* vehicle, 19445 ± 6774 pg/ml *vs.* 20295 ± 6610 pg/ml, p=0.93), plasma ACTH levels after CRH stimulation were significantly lower in the bevacizumab group than in the vehicle group (4.6 ± 3.0 pg/ml *vs.* 34.5 ± 11.7 pg/ml, p=0.035). We infer that angiogenesis is important for engrafted-POs functionality in ISWAT (**Figure 4F**).

## Discussion

This study indicated that hESC-derived POs could be engrafted into and function in the subcutaneous tissue of hypopituitary SCID mice. The transplanted mice responded to CRH with release of ACTH into the circulation, indicating that injected CRH stimulated the transplanted ACTH-producing cells. Comparisons between ISWAT and AR transplantation indicated that vessel-rich subcutaneous adipose tissue is better than the relatively vessel-poor potential space beneath back skin, yielding more persistent ACTH secretion. PSC-derived POs transplantation improves physical activity levels and body weight (6, 8); our work confirmed this. Moreover, hESC-derived POs express angiogenic factors such as *VEGF* and *ANGPT2*, suggesting that POs autonomously promote vascularization and engraftment. Levels of *VEGFC* and *ANGPT2* expression rose in POs after transplantation; perhaps adiponectin, a cytokine released from adipocytes, contributed to this (22). Finally, ACTH secretion was reduced by bevacizumab administration, indicating that angiogenesis is important, at least in subcutaneous transplantation. Peri-implant vascularity could be important for engraftment. Perhaps relatively high ACTH secretion observed in some AR-group mice reflected serendipitous proximity to blood vessels, resulting in successful PO engraftment. Of weight here is that the subcutaneous vascular plexus is abundant in the adipose tissue layer (23). Humoral factors from adipose tissue such as adiponectin may contribute by supporting angiogenesis. These results and considerations support intra-adipose tissue implantation of hESC-derived POs if a subcutaneous site is selected.

Demonstration of function in subcutaneously transplanted PSC-derived POs is an important step in regenerative-medicine technology. Kidney subcapsular transplantation and subcutaneous transplantation differ importantly. POs recipient patients suffer from hypopituitarism and thus are sensitive to stresses such as invasive procedures. Both kidney subcapsular and subcutaneous transplantation require general anesthesia, but in humans, subcutaneous transplantation can be performed under local anesthesia, substantially less invasive than the alternative. Kidney subcapsular transplantation could damage a normal kidney. Risks of collateral damage during subcutaneous transplantation are by contrast low; the surgery itself is easy and can be done quickly, in mouse or in human, perhaps permitting outpatient work. Important in transplantation of cells derived from PSC is graft removal if tumor develops. Subcutaneous transplantation allows relatively simple and non-invasive removal.

That transplantation into adipose tissue is more effective than transplantation into an avascular site is important for clinical application. PSC-derived pancreatic-endoderm cells engrafted successfully in the deep subcutaneous tissues of the abdominal wall of type 1 diabetes human patients (24, 25). Our success is consonant with theirs, supporting the merits of adipose tissue endografts.

Mouse ACTH secretion varied. The reasons for the variation include: 1) ACTH secretion in reaction to stressors (a little stress during sample collection can affect ACTH values), 2) hemolysis, and 3) large gaps in results caused by small measurement errors due to minute sample volume. Although at no blood collection point did differences achieve statistical significance, the transplant group tended to secrete higher levels of ACTH than the sham group. This has the potential to prevent adrenal crisis, which is an important goal of pituitary regenerative medicine.

To evaluate adrenal function, levels of ACTH and corticosterone, a hormone regulated by ACTH, must be determined simultaneously. In this study, however, we selected blood collection from the tail to permit repeated sampling. That sample volumes were very small precluded measurement of both ACTH and corticosterone at the same time. However, corticosterone values increased on CRH loading after transplantation of POs, although these were placed in a renal subcapsular site and the mice were decapitated (6, 8).

On histologic study, POs after subcutaneous transplantation differ from POs *in vitro*; they are soft and they collapse, losing their original appearance. More pituitary cells are seen in **Figure 3**, showing POs transplanted nearly 200d after differentiation, than in **Supplementary Figure 1**: The more time *in vitro*, the more differentiated (7).

Subcutaneous transplantation of POs increased ACTH secretion and improved physical activity in hypopituitary mice but without equaling normal mice and with some weight loss. How many POs are required to normalize plasma ACTH levels awaits study. Our methods of inducing differentiation of POs from PSCs generate ACTH-producing cells efficiently, with fewer cells dedicated to production of other adenohypophyseal hormones (data not shown). We speculate that growth hormone deficiency (26) and central hypogonadotropic hypogonadism (27) contributed to decreased activity and to weight loss.

Since we studied SCID mice, we did not investigate immune responses. Our follow-up work will focus on treatment of “wild-type” mice with hypopituitarism, addressing the utility of immunosuppression or of HLA-editing iPSC-derived POs (28). Solving this issue will bring us closer to clinical application in humans.

## Conclusion

We indicated that hESC-derived POs function following subcutaneous transplantation in mice. An appropriate site for subcutaneous transplantation is adipose tissue, which is richly vascularized. Angiogenesis is important for subcutaneous engraftment of hESC-derived POs.

## Data availability statement

The original contributions presented in the study are included in the article/**Supplementary Material**. Further inquiries can be directed to the corresponding author.

## Ethics statement

The animal study was reviewed and approved by Animal Experimentation Committee of the Nagoya University Graduate School of Medicine.

## Author contributions

Authorship: Participation included writing of the article, HSa and HSu. Research design, HSa, HSu, KT, YN, HH, TK, EI, TM, ST, AK, TN, HA, and RS. Performance of the research, HSa, HSu, SM, MSa, MSo, TM, TA, and HO. Data analysis, HSa and ST. All authors contributed to the article and approved the submitted version.
