# Supplementary figures and images for: Subcutaneous transplantation of human embryonic stem cells-derived pituitary organoids
Source: Front Endocrinol (Lausanne). 2023 Mar 2;14:1130465. doi: 10.3389/fendo.2023.1130465 (PMC10018142; doi:10.3389/fendo.2023.1130465)

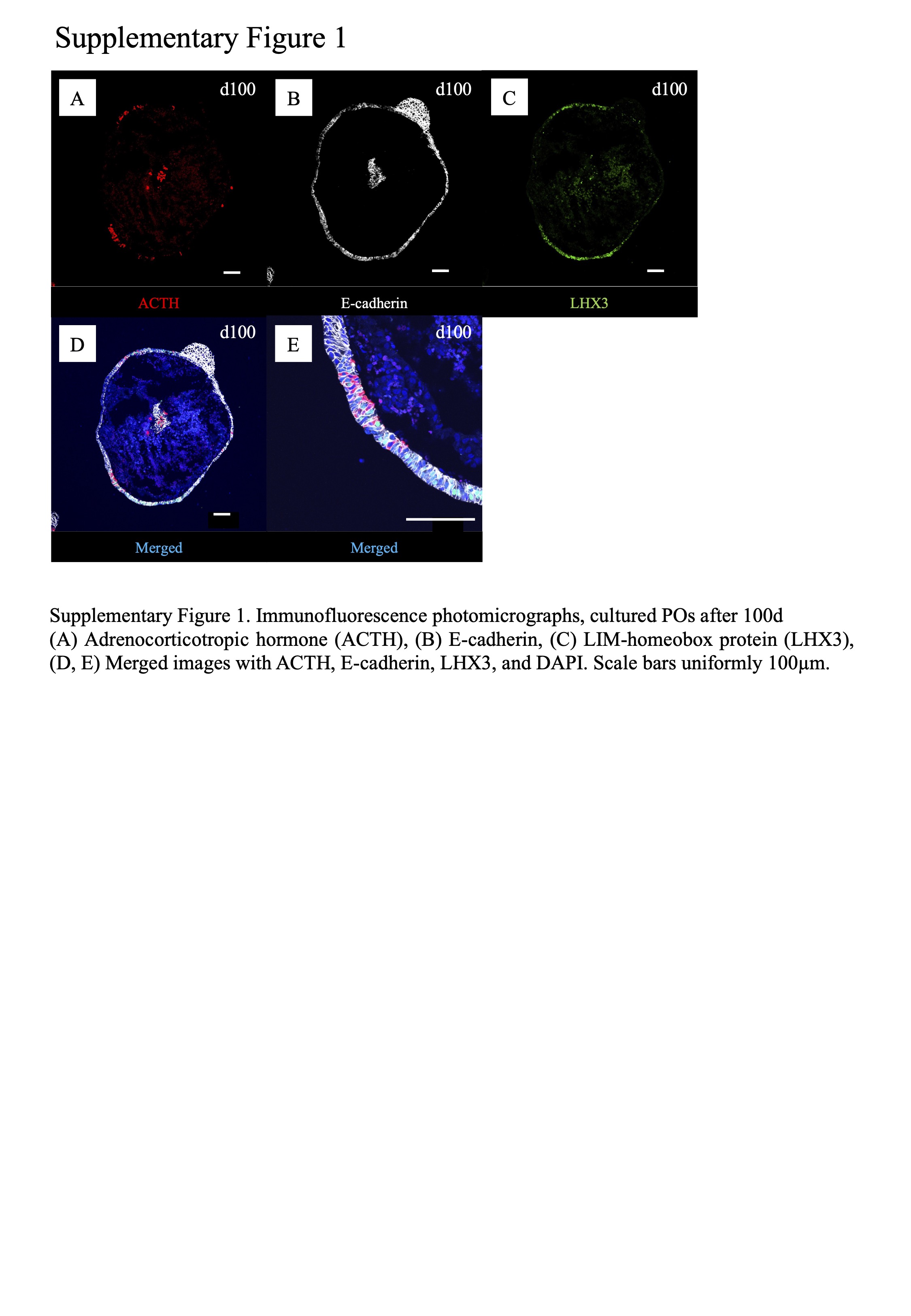

Supplement: Supplementary file 1 [file Image_1.jpeg]

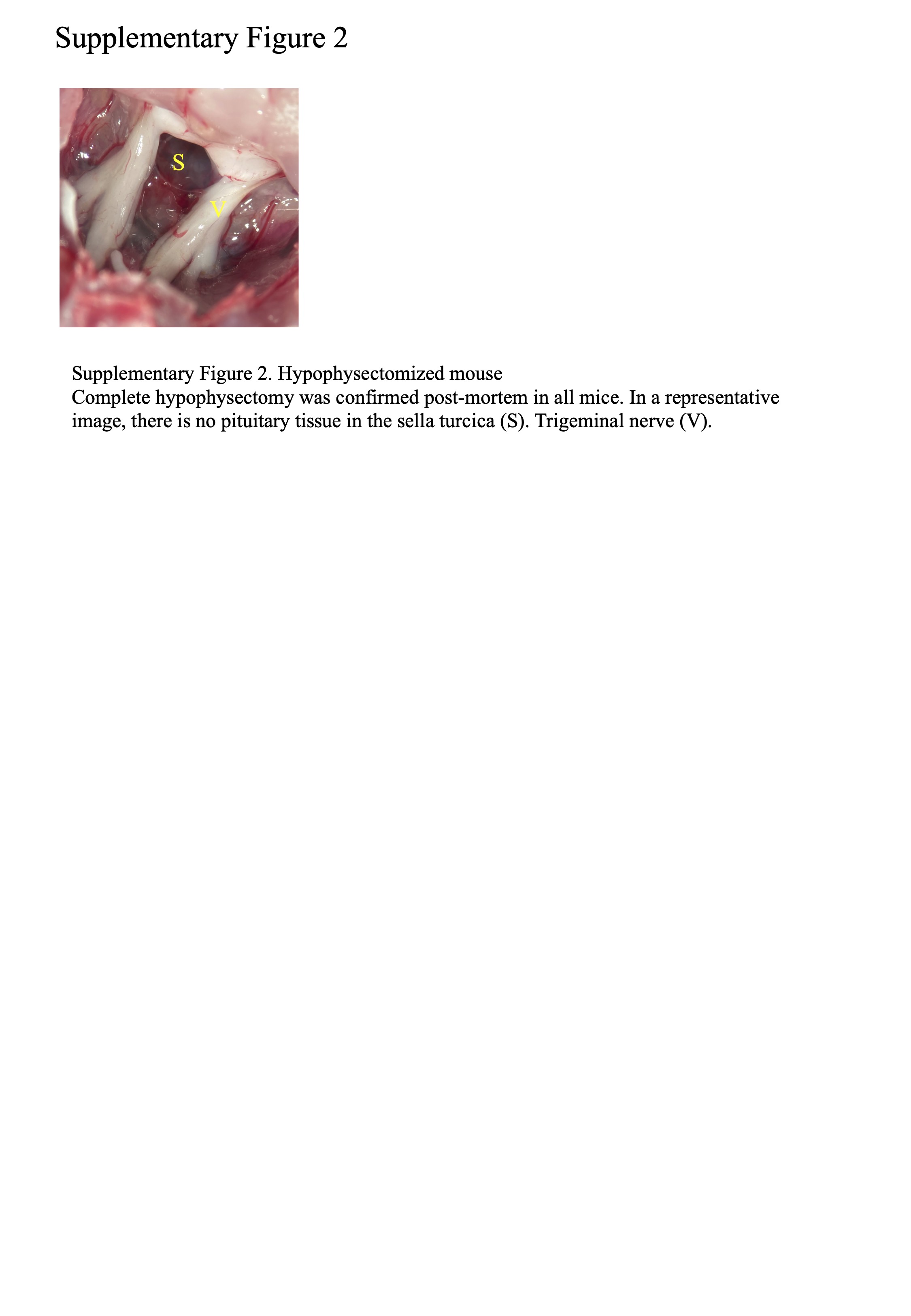

Supplement: Supplementary file 2 [file Image_2.jpg]

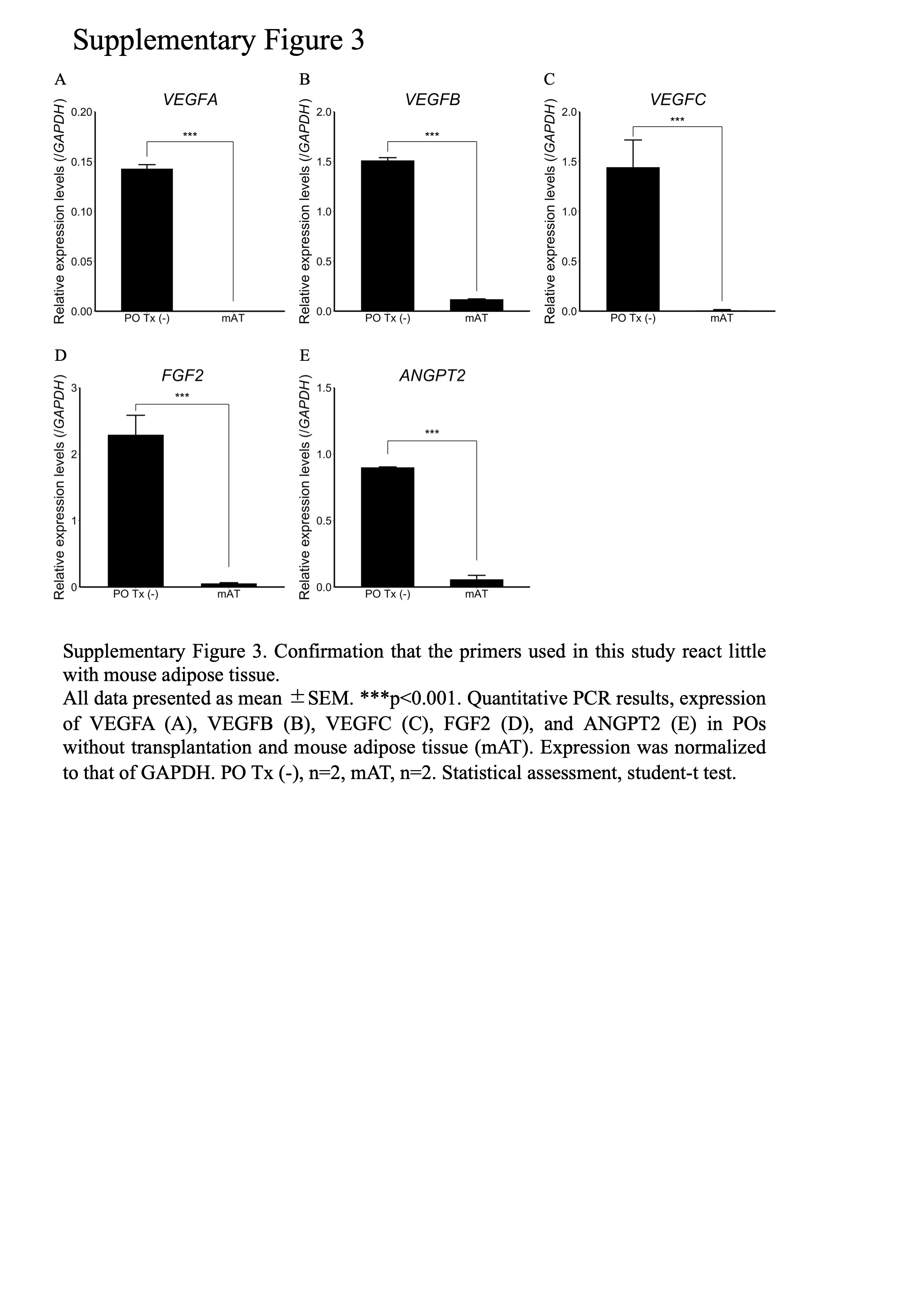

Supplement: Supplementary file 3 [file Image_3.jpg]

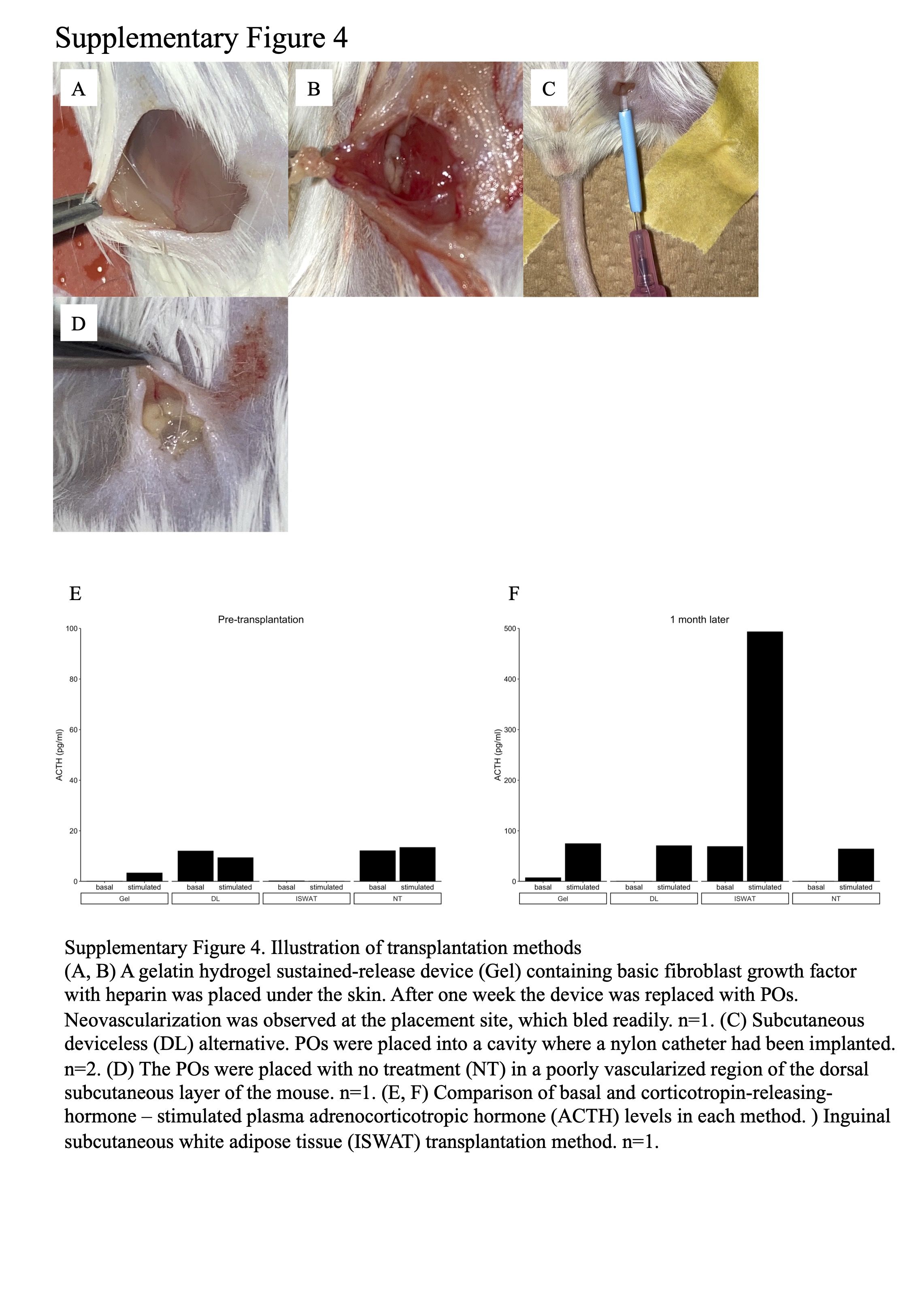

Supplement: Supplementary file 4 [file Image_4.jpg]

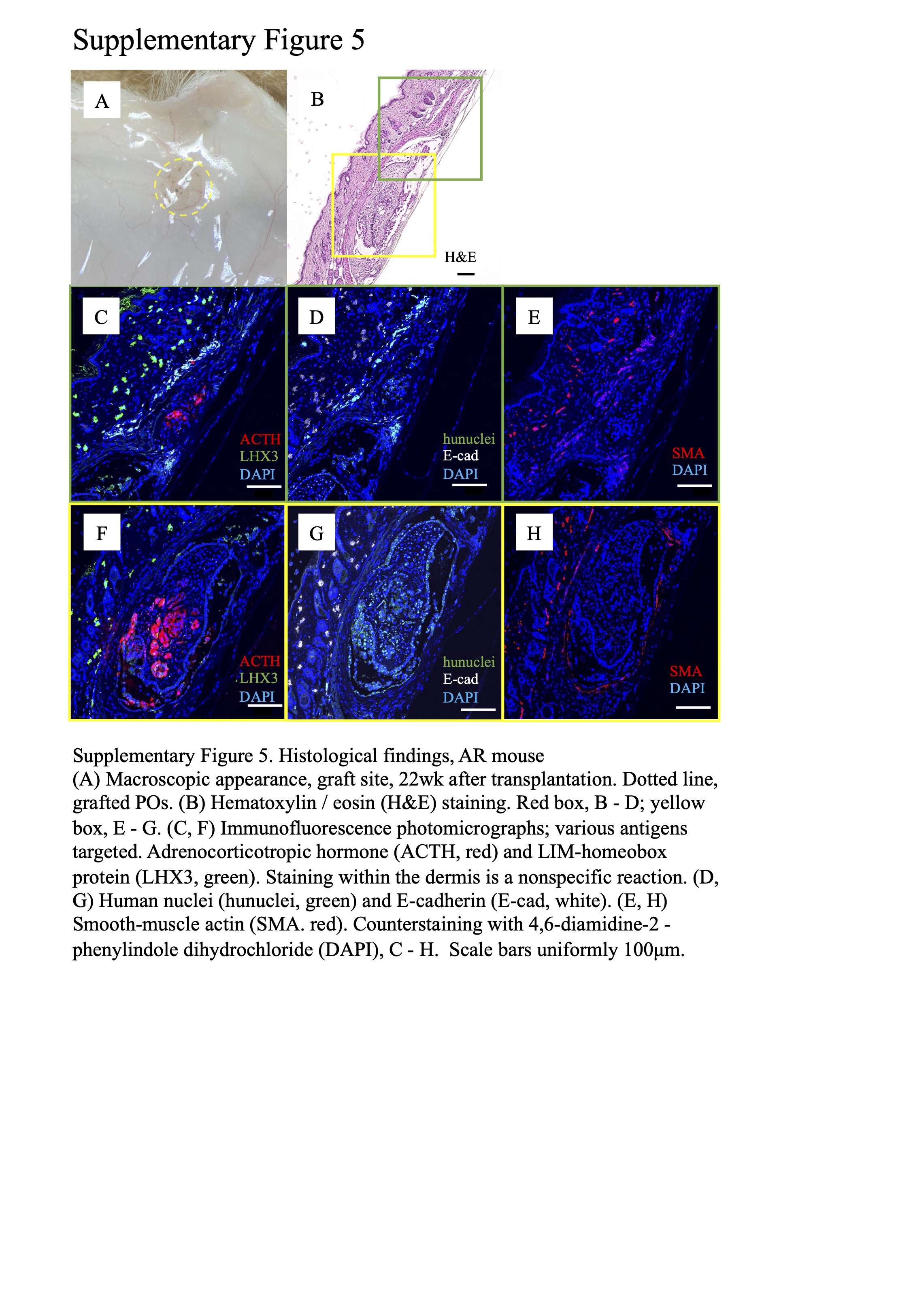

Supplement: Supplementary file 5 [file Image_5.jpg]
